# Serum levels of interleukin-22, cardiometabolic risk factors and incident type 2 diabetes: KORA F4/FF4 study

**DOI:** 10.1186/s12933-017-0498-6

**Published:** 2017-01-31

**Authors:** Christian Herder, Julia M. Kannenberg, Maren Carstensen-Kirberg, Cornelia Huth, Christa Meisinger, Wolfgang Koenig, Annette Peters, Wolfgang Rathmann, Michael Roden, Barbara Thorand

**Affiliations:** 1 Institute for Clinical Diabetology, German Diabetes Center, Leibniz Center for Diabetes Research at Heinrich Heine University Düsseldorf, Auf’m Hennekamp 65, 40225 Düsseldorf, Germany; 2 German Center for Diabetes Research (DZD), München-Neuherberg, Germany; 3 Institute of Epidemiology II, Helmholtz Zentrum München, German Research Center for Environmental Health, Neuherberg, Germany; 4 Deutsches Herzzentrum München, Technische Universität München, Munich, Germany; 5 German Center for Cardiovascular Research (DZHK), Partner Site Munich Heart Alliance, Munich, Germany; 6 Institute for Biometrics and Epidemiology, German Diabetes Center, Leibniz Center for Diabetes Research at Heinrich Heine University Düsseldorf, Düsseldorf, Germany; 7 Department of Endocrinology and Diabetology, Medical Faculty, University Hospital Düsseldorf, Düsseldorf, Germany

**Keywords:** Inflammation, Interleukin-22, Cytokine, Type 2 diabetes, Cardiometabolic risk, Cohort study

## Abstract

**Aims:**

Interleukin-22 (IL-22) has beneficial effects on body weight, insulin resistance and inflammation in different mouse models, but its relevance for the development of type 2 diabetes in humans is unknown. We aimed to identify correlates of serum IL-22 levels and to test the hypothesis that higher IL-22 levels are associated with lower diabetes incidence.

**Methods:**

Cross-sectional associations between serum IL-22, cardiometabolic risk factors and glucose tolerance status were investigated in 1107 persons of the population-based KORA F4 study. The prospective association between serum IL-22 and incident type 2 diabetes was assessed in 504 initially non-diabetic study participants in both the KORA F4 study and its 7-year follow-up examination KORA FF4, 76 of whom developed diabetes.

**Results:**

Male sex, current smoking, lower HDL cholesterol, lower estimated glomerular filtration rate and higher serum interleukin-1 receptor antagonist were associated with higher IL-22 levels after adjustment for confounders (all *P* < 0.05). Serum IL-22 showed no associations with glucose tolerance status, prediabetes or type 2 diabetes. Baseline serum IL-22 levels (median, 25th/75th percentiles) for incident type 2 diabetes cases and non-cases were 6.28 (1.95; 12.35) and 6.45 (1.95; 11.80) pg/ml, respectively (age and sex-adjusted *P* = 0.744). The age and sex-adjusted OR (95% CI) per doubling of IL-22 for incident type 2 diabetes of 1.02 (0.85; 1.23) was almost unchanged after consideration of further confounders.

**Conclusions:**

High serum levels of IL-22 were positively rather than inversely associated with several cardiometabolic risk factors. However, these associations did not translate into an increased risk for type 2 diabetes. Thus, our data argue against the utility of IL-22 as biomarker for prevalent or incident type 2 diabetes in humans, but identify potential determinants of IL-22 levels which merits further research in the context of cardiovascular diseases.

**Electronic supplementary material:**

The online version of this article (doi:10.1186/s12933-017-0498-6) contains supplementary material, which is available to authorized users.

## Background

Interleukin-22 (IL-22), a member of the IL-10 cytokine family, is produced by different leukocyte subsets and signals through a heterodimer of the IL-22 receptor 1 (IL-22R1) paired with IL-10R2. While the latter is widely expressed, expression of IL-22R1 is restricted mainly to cell types in the pancreas and epithelial cells in liver, intestine, kidney and skin, which determines the specificity of IL-22 action [1, 2].

IL-22 represents a crucial regulator of gut epithelial barrier integrity and thereby prevents peripheral dissemination of commensal bacteria and limits systemic inflammation [3]. A recent study extended these data to metabolic disorders and found that mice lacking IL-22 signaling are prone to obesity and insulin resistance [4]. Several mouse models of obesity also revealed beneficial effects of IL-22 treatment on glucose homeostasis, insulin sensitivity, insulin secretion and inflammation [4, 5]. Other studies have challenged the notion that IL-22 ameliorates insulin resistance and chronic inflammation in mice [6] or humans [7, 8].

To date, it remains unclear how IL-22 is regulated in humans, which physiological role circulating IL-22 levels exert and whether endogenous IL-22 levels are associated with protection against obesity and related disorders. Two cross-sectional studies reported higher rather than lower systemic levels of IL-22 in patients with type 2 diabetes and in patients with coronary artery disease from hospital-based samples than in healthy controls [9, 10], but population-based data or prospective studies on IL-22 and cardiometabolic diseases are not available.

Therefore, the aims of this study were (1) to identify correlates of IL-22 serum levels within a population-based cohort and (2) to test the hypothesis (based on the aforementioned mechanistic studies) that higher serum IL-22 levels associate with lower incidence of type 2 diabetes.

## Study participants and methods

### Study population

Data are based on the Cooperative Health Research in the Region of Augsburg (KORA) F4 study (2006–2008) and the KORA FF4 study (2013–2014), both follow-up examinations of the population-based KORA S4 study (1999–2001) conducted in Augsburg (Germany) and two surrounding counties. The design of the KORA studies has been described before [11].

All study participants without known diabetes were assigned to receive a standard 75-g oral glucose tolerance test (OGTT) in both KORA F4 and KORA FF4. Glucose tolerance categories were defined using fasting and 2-h glucose levels according to the 2003 American Diabetes Association criteria [12]. Prediabetes was defined as IFG, IGT or combined IFG/IGT. Previously known type 2 diabetes was defined as self-report that could be validated by the responsible physician, or as current use of antidiabetic agents. Incident type 2 diabetes in KORA FF4 was defined based on a self-reported diagnosis of type 2 diabetes since KORA F4 that could be validated, current use of antidiabetic agents, or fasting and/or 2-h glucose levels in the OGTT above the aforementioned criteria.

This study included all persons aged 62–81 years in KORA F4 (*n* = 1161). For the cross-sectional study part we excluded persons with unclear glucose tolerance status due to missing values for fasting and/or 2-h glucose (*n* = 26), type 1 diabetes (*n* = 2) or other missing covariables (*n* = 26), resulting in a sample size of 1107 individuals.

The prospective part used all of these participants, for whom follow-up data from KORA FF4 were available (*n* = 641). Baseline characteristics of the drop-outs from the cross-sectional analysis sample (*n* = 1107) who did not participate in KORA FF4 (*n* = 466) are summarised in Additional file 1: Table S1. Reasons for non-participation included the following: individuals had died, refused/were too ill/were not interested or too busy to participate, or could not be contacted. Since incident type 2 diabetes was studied as outcome we excluded participants with known (*n* = 76) or newly diagnosed (*n* = 39) type 2 diabetes in KORA F4, with unclear glucose tolerance status due to missing values for fasting and/or 2-h glucose (*n* = 21) or with other missing covariables (*n* = 1), which left a sample size of 504 individuals.

The assessment of anthropometric and metabolic variables and of lifestyle factors was performed as described [11, 13]. Hypertension was defined as blood pressure of 140/90 mmHg or higher, or antihypertensive medication given that the subjects were aware of being hypertensive. The estimated glomerular filtration rate (eGFR) was calculated using the chronic kidney disease epidemiology (CKD-EPI) creatinine equation [14]. Study participants were classified as physically active if they reported >1 h sports/week in summer and in winter.

### Quantification of IL-22 serum levels

IL-22 levels were assessed in serum samples that had been continuously stored at −80 °C between blood sampling and analysis, which was performed between 08/2015 and 10/2015. Measurements were carried out using the Quantikine ELISA (R&D Systems, Wiesbaden, Germany). The limit of detection (LOD) was 3.9 pg/ml. Measurements below the LOD for 347 sera were assumed to be evenly distributed between 0 and the LOD and therefore assigned a value of 0.5*LOD as in previous analyses within the KORA cohort [15, 16]. Values > 99th percentile (extreme outliers between 74.4 and 406 pg/ml, *n* = 11) were assigned the IL-22 level of the 99th percentile (74.4 pg/ml). Intra- and inter-assay CV were 5.5 and 9.3%, respectively.

### Statistical analysis

Associations between serum IL-22 and other variables of the KORA F4 sample were described by baseline characteristics stratified by quarters of IL-22 levels and age and sex-adjusted *P* values from linear regression analysis. Due to the high number of samples with IL-22 below the LOD, quarter sizes were unequal with quarter 1 (comprising all individuals with IL-22 below the LOD) being larger than quarters 2–4 (comprising all individuals with measurable IL-22 levels).

Independent correlates of IL-22 levels at baseline were identified by multivariable linear regression analysis employing a forward selection of variables and exclusion of variables without significant association (*P* > 0.05) with serum ln (IL-22). We added the following covariables: step 1, age and sex; step 2, smoking status (current/former/never), alcohol consumption, physical activity (active/inactive); step 3, BMI; step 4, HDL cholesterol, LDL cholesterol, triglycerides, hypertension (yes/no), history of myocardial infarction (yes/no), eGFR; step 5, circulating levels of high-sensitivity C-reactive protein (hsCRP), IL-6, IL-18, tumour necrosis factor (TNF)α, IL-1 receptor antagonist (IL-1RA), soluble intercellular adhesion molecule-1 (sICAM-1), adiponectin.

Cross-sectional associations of ln(IL-22) with single glucose tolerance status groups or with prediabetes/diabetes were estimated by multinomial logistic regression with normal glucose tolerance as reference and using the following predefined set of potential confounders from previous KORA analyses [11]: model 1, adjusted for age and sex; model 2, model 1 + smoking, alcohol consumption, physical activity; model 3, model 2 + BMI; model 4, model 3 + HDL cholesterol, LDL cholesterol, triglycerides, hypertension, prevalent myocardial infarction, eGFR. Age, alcohol consumption, BMI, HDL cholesterol, LDL cholesterol, triglycerides and eGFR entered the models as continuous variables.

The association between a doubling in serum IL-22 as continuous variable and incident type 2 diabetes in the prospective analysis was assessed by multivariable logistic regression using the same set of covariables as in the cross-sectional analysis. We performed a test of linearity in IL-22 using restricted cubic splines functions with k = 3, 4, 5 equally spaced knots between the 0.05 and 0.95 quantiles for the fully adjusted model 4 using the ‘rms’ package in R. Potential sex differences were assessed using an interaction term IL-22*sex in the logistic regression models.

We performed the following sensitivity analyses: (1) analysis of the original dataset before replacement of outliers with IL-22 levels corresponding to the 99th percentile; (2) analysis of the data treating IL-22 as dichotomous exposure (IL-22 levels above versus below the LOD); (3) analysis restricted to current non-smokers.

All statistical analyses were performed with R version 3.2.4 (R Core Team, R Foundation for Statistical Computing, Vienna, Austria). A *P* value <0.05 was considered to indicate statistical significance.

## Results

### Associations of serum IL-22 levels with cardiometabolic risk factors

In the cross-sectional analysis based on 1107 individuals, IL-22 serum levels were higher in men than in women (distributions shown in Additional file 1: Figure S1) and positively associated with age (Table 1). In age and sex-adjusted analyses, IL-22 serum levels were positively associated with BMI, fasting and 2-h insulin, HOMA-IR, smoking status and circulating levels of IL-18, IL-1RA and sICAM-1, but negatively with total cholesterol, HDL cholesterol and estimated glomerular filtration rate (eGFR) (Table 1).

**Table 1:** Description of the KORA F4 study population stratified by quarters of IL-22 concentrations (cross-sectional analysis)

Variable	Quarter 1	Quarter 2	Quarter 3	Quarter 4	*P* ^a^
*n*	347	208	277	275	
IL-22 (pg/ml; range)	1.95–1.95	3.90–6.74	6.78–13.33	13.36–74.39	
Age (years)	69.6 ± 5.3	70.2 ± 5.5	70.3 ± 5.1	70.9 ± 5.7	0.002
Sex (% male)	31.7	48.6	57.4	73.1	<0.001
BMI (kg/m^2^)	28.2 ± 4.6	28.7 ± 4.5	29.5 ± 4.1	28.7 ± 4.5	0.007
Fasting glucose (mmol/l)^b^	5.45 ± 0.85	5.49 ± 0.62	5.56 ± 0.65	5.50 ± 0.62	0.483
2-h glucose (mmol/l)^b^	7.05 ± 2.28	7.13 ± 2.25	7.31 ± 2.39	7.04 ± 2.29	0.697
Fasting insulin (pmol/l)^b^	29.1 (19.8; 45.8)	27.0 (19.8; 43.5)	31.8 (21.0; 64.5)	31.5 (21.0; 57.6)	<0.001
2-h insulin (pmol/l)^b^	307.8 (175.8; 474.0)	319.8 (156.0; 506.2)	372.6 (219.0; 597.6)	321.3 (192.3; 583.4)	0.004
HOMA-IR^b^	1.17 (0.75; 1.92)	1.11 (0.79; 1.80)	1.31 (0.81; 2.71)	1.24 (0.80; 2.49)	<0.001
ISI (composite) (1/((mmol/l)x(pmol/l)))^b^	18.0 (10.4; 27.5)	17.7 (10.4; 32.4)	14.4 (8.6; 24.5)	16.5 (9.0; 27.4)	0.869
HbA1c (%)	5.61 ± 0.47	5.60 ± 0.35	5.65 ± 0.34	5.63 ± 0.38	0.419
HbA1c (mmol/mol)	38 ± 5	38 ± 4	38 ± 4	38 ± 4	0.419
Glucose tolerance status: NGT/IFG/IGT/IFG&IGT/ndT2D/T2D (%)	43.8/16.1/12.1/11.5/5.2/11.2	41.3/19.2/8.7/11.1/7.7/12.0	34.7/22.0/8.7/9.7/7.9/17.0	36.7/18.5/9.1/12.0/5.8/17.8	0.766
Hypertension (%)	59.4	56.5	67.0	67.0	0.628
Total cholesterol (mmol/l)^c^	6.13 ± 1.05	5.95 ± 0.99	5.84 ± 0.99	5.69 ± 0.96	0.002
LDL cholesterol (mmol/l)^c^	3.88 ± 0.95	3.84 ± 0.86	3.81 ± 0.88	3.63 ± 0.88	0.072
HDL cholesterol (mmol/l)^c^	1.56 ± 0.38	1.48 ± 0.34	1.37 ± 0.35	1.35 ± 0.36	<0.001
Triglycerides (mmol/l)^c^	1.26 (0.91; 1.74)	1.22 (0.92; 1.64)	1.31 (1.01; 1.85)	1.30 (0.91; 1.84)	0.635
Use of lipid-lowering drugs (%)	24.2	23.2	26.4	23.8	0.865
eGFR (ml/min per 1.73 m^2^)	79.7 ± 12.8	77.3 ± 13.8	74.9 ± 14.8	73.3 ± 17.7	<0.001
eGFR < 60 ml/min per 1.73 m^2^) (%)	6.9	13.9	16.6	21.8	<0.001
Smoking (never/former/current) (%)	61.4/35.4/3.2	53.8/38.0/8.2	47.5/44.2/8.3	38.6/49.3/12.1	<0.001
Physically active (%)	53.6	51.4	49.6	44.5	0.174
hs C-reactive protein (mg/l)	1.44 (0.70; 2.87)	1.38 (0.72; 2.91)	1.59 (0.87; 3.21)	1.94 (0.89; 3.90)	0.310
IL-6 (pg/ml)	1.43 (0.93; 2.28)	1.52 (1.03; 2.12)	1.70 (1.27; 2.51)	1.92 (1.33; 2.92)	0.586
IL-18 (pg/ml)	296.0 (228.0; 379.0)	308.0 (251.5; 412.0)	344.5 (269.2; 433.8)	342.5 (263.8; 451.2)	0.032
Tumour necrosis factor α (pg/ml)	1.91 (1.40; 2.77)	1.84 (1.38; 2.42)	2.18 (1.61; 3.17)	2.22 (1.63; 3.16)	0.305
IL-1 receptor antagonist (pg/ml)	295.7 (224.1; 393.3)	289.1 (235.0; 384.1)	326.2 (247.3; 432.2)	323.1 (248.4; 446.1)	<0.001
Soluble intercellular adhesion molecule 1 (ng/ml)	228.4 ± 53.9	235.9 ± 49.9	241.8 ± 58.0	246.1 ± 67.7	<0.001
Adiponectin (µg/ml)	11.62 (8.13; 17.25)	9.87 (6.79; 14.82)	8.82 (5.68; 13.8)	9.30 (5.94; 12.88)	0.311

Data are given as mean ± SD, median and 25th; 75th percentiles or percentages, unless indicated otherwise. The age and sex-adjusted *P* values are from linear regression analysis (likelihood ratio tests comparing models with ln IL-22 as dependent variable and the respective variable, age and sex as independent variables to models with age and sex as independent variables only). The analysis for age is adjusted for sex only, the analysis for sex is adjusted for age only

eGFR, estimated glomerular filtration rate; hs, high-sensitivity; IFG, impaired fasting glucose; IGT, impaired glucose tolerance; ISI, insulin sensitivity index; ndT2D, newly diagnosed type 2 diabetes; NGT, normal glucose tolerance; T2D, type 2 diabetes

^a^Adjusted for age and sex

^b^Individuals with known type 2 diabetes excluded (*n* = 160)

^c^Individuals using lipid-lowering drugs excluded (*n* = 270)

Multivariable linear regression analysis identified male sex, current smoking, lower HDL cholesterol, lower eGFR and higher serum IL-1RA as independent correlates of higher IL-22 (Table 2).

**Table 2:** Correlates of IL-22 levels at baseline (cross-sectional analysis): final model from multivariable linear regression analysis

Variable	*β* (95% CI)	*P*
Male sex	0.524 (0.401; 0.648)	<0.001
Current smoking	0.439 (0.228; 0.651)	<0.001
Former smoking	0.060 (−0.061; 0.181)	0.332
HDL cholesterol (mmol/l)	−0.172 (−0.337; −0.008)	0.041
eGFR (ml/min per 1.73 m^2^)	−0.010 (−0.014; −0.007)	<0.001
IL-1RA (pg/ml)	0.001 (0.000; 0.001)	<0.001

Regression coefficients *β* and corresponding 95% confidence intervals (per given unit in the table) and *P* values are based on a final model with ln(IL-22) as dependent variable after a forward selection including blocks of independent variables at each step (see below). At each step non-significant variables (*P* > 0.05) were excluded from further analysis

*Step 1* age and sex

*Step 2* smoking status, alcohol consumption, physical activity

*Step 3* BMI

*Step 4* HDL cholesterol, LDL cholesterol, triglycerides, hypertension, history of myocardial infarction, eGFR

*Step 5* hsCRP, IL-6, IL-18, TNFα, IL-1RA, sICAM-1, adiponectin

eGFR, estimated glomerular filtration rate; IL-1RA, IL-1 receptor antagonist

However, serum IL-22 did neither differ between groups with different glucose tolerance status (Additional file 1: Table S2) nor between individuals with normal glucose tolerance, prediabetes (IFG and/or IGT) or type 2 diabetes (Additional file 1: Table S3) at all levels of adjustment.

### No association between serum IL-22 levels and incident type 2 diabetes

As shown in Additional file 1: Table S1, individuals without follow-up data (i.e. non-participants in KORA FF4) were older, had a higher BMI and a less favourable cardiometabolic risk profile compared to individuals who participated in KORA FF4. Importantly, serum IL-22 did not differ between both groups.

The prospective part of the study was based on 504 individuals who were free of diabetes in the KORA F4 survey and for whom all relevant data from the follow-up KORA FF4 study were available.

Of these, 76 individuals developed type 2 diabetes, whereas 428 individuals remained diabetes-free. Baseline levels of serum IL-22 levels (median and 25th/75th percentiles) were 6.45 (1.95; 11.80) pg/ml for cases and 6.28 (1.95; 12.35) pg/ml for non-cases (age and sex-adjusted *P* = 0.744, Additional file 1: Table S4), which was reflected by an OR (95% CI) per doubling of serum IL-22 (i.e. twofold increase in baseline IL-22) for incident type 2 diabetes of 1.02 (0.85; 1.23) (Table 3). Addition of further covariables to the model had virtually no impact on the results (Table 3). We did not detect any indication for non-linearity (Wald statistics: *P* = 0.524 for *k* = 3 knots, *P* = 0.549 for *k* = 4 knots, *P* = 0.714 for *k* = 5 knots for the fully adjusted model 4). We did not observe a significant interaction by sex for this association (*P*_interaction_ between 0.07 and 0.14 for models 1–4).

**Table 3:** OR (95% CI) for incident type 2 diabetes per doubling in serum IL-22 (prospective analysis)

Model	Covariables	OR (95% CI)	*P*
1	Age, sex	1.02 (0.84; 1.23)	0.838
2	Model 1 + current smoking, former smoking, alcohol consumption, physical activity	1.04 (0.86; 1.26)	0.703
3	Model 2 + BMI	1.01 (0.83; 1.24)	0.893
4	Model 3 + HDL cholesterol, LDL cholesterol, triglycerides, use of lipid-lowering drugs, hypertension, prevalent myocardial infarction, estimated glomerular filtration rate	1.03 (0.83; 1.27)	0.797

Our sensitivity analyses showed almost identical results (1) when we used the original dataset before replacement of outliers with IL-22 levels corresponding to the 99th percentile; (2) when we treated IL-22 as dichotomous exposure (IL-22 levels above versus below the LOD) and (3) when we restricted our analysis to non-smokers (Additional file 1: Table S5).

## Discussion

The main findings of this study are that (1) serum IL-22 exhibited positive rather than negative associations with multiple cardiometabolic risk factors of type 2 diabetes and related complications and (2) IL-22 levels were neither associated with glucose tolerance and diabetes status nor with incident type 2 diabetes during a 7-year follow-up period.

### IL-22 and cardiometabolic risk factors

Our data are novel because previous studies on IL-22 and metabolic disorders mainly focused on mouse models, whereas research in humans is warranted to assess the potential role of IL-22 as biomarker for diabetes risk or as candidate for treatment studies [17]. This study extends the current literature by demonstrating that male sex, current smoking, lower HDL cholesterol, lower eGFR and higher IL-1RA were associated with higher IL-22 levels independently of confounders.

It can be speculated that sex hormones are regulators of IL-22, but data from experimental or other epidemiological studies in this context are not available. The positive association between current smoking and higher IL-22 levels is in line with proinflammatory effects of smoking that have been demonstrated in mechanistic studies and that are reflected by a systemic upregulation of numerous other biomarkers of inflammation [18]. Due to the cross-sectional design of the study the causal directions in the associations between higher IL-22 on the one hand and lower HDL cholesterol, lower eGFR and higher IL-1RA on the other hand cannot be determined. However, there is evidence from other experimental settings that may be relevant for a better understanding of our observations. First, IL-22 has been demonstrated to downregulate the expression of ATP-binding cassette sub-family G member 1 (ABCG1) and to reduce cholesterol efflux in macrophages, which may link IL-22 with decreased HDL cholesterol levels and increased cardiovascular risk [19]. Second, IL-22 has been reported to have both beneficial and detrimental effects depending on the type of experimental model of kidney injury [2, 20]. Data from other human cohorts are not available, so that further studies are necessary to confirm the inverse association between IL-22 and eGFR and to explore the relevance of this association for the development of chronic kidney disease in older individuals. Third, IL-22 has been implicated in the increased production of IL-1RA mediated by protein kinase C δ (PKCδ) and NLR family CARD domain-containing protein 4 (NLRC4) [21], which suggests that IL-22 could indeed contribute to higher IL-1RA levels. An alternative explanation is related to the fact that the proinflammatory cytokine IL-1β is not only a known positive regulator of its endogenous inhibitor IL-1RA, but also of IL-22 [1, 2, 7], so that the association found in our cohort could as well reflect the result of a joint upregulation of both proteins downstream of IL-1β.

In addition, we found positive associations with age, BMI, fasting and 2-h insulin, HOMA-IR, IL-18 and sICAM-1. These were, however, not significant after extensive adjustment, but may also link higher IL-22 levels with higher risk of cardiometabolic diseases.

We observed that the dynamic range of IL-22 in the circulation appears to span at least two orders of magnitude in the general population with a large proportion of individuals showing very low levels (i.e. below our LOD of 3.9 pg/ml). This could indicate that IL-22 levels are tightly regulated in the absence of appropriate stimuli. However, the demographic, anthropometric and metabolic characteristics of this subgroup did not differ very strongly from the remainder of the cohort, so that the precise reason for the broad distribution of circulating IL-22 needs further research.

Taken together, our study provides novel data on the relationships between IL-22 levels and sex, smoking, lipid metabolism, IL-1β-related processes and kidney function that merit further studies. Importantly, the data suggest that *higher* endogenous circulating IL-22 may be related to *higher* levels of several cardiometabolic risk factors, which was not expected given the anti-inflammatory and anti-diabetic effects observed in mouse models [3–5].

### IL-22 and type 2 diabetes

Despite the link between higher IL-22 levels and the more pronounced cardiometabolic risk profile discussed above, it seems that these associations had no detectable impact on glucose tolerance and diabetes status. Although we observed positive associations of IL-22 with fasting and 2-h insulin levels and with HOMA-IR after adjustment for age and sex, they were not significant anymore after further adjustment for confounding factors.

Previous clinical studies found that plasma levels of IL-22 and the number of IL-22-producing CD4^+^ T cells were higher in insulin-resistant or type 2 diabetic obese individuals than in insulin-sensitive obese or lean individuals [7, 8], but results were based on very small samples. In addition, analyses were not adjusted for any covariables, which represents an important limitation given large differences in HDL cholesterol levels between groups in one of the aforementioned studies [7] and given our findings that sex, smoking, HDL cholesterol, inflammation and kidney function should be considered as potential confounders in any analyses of circulating IL-22 levels.

In line with these findings, one hospital-based study reported higher plasma IL-22 levels in patients with type 2 diabetes than in healthy controls or individuals with metabolically healthy obesity [9]. A second hospital-based study observed higher serum IL-22 concentrations in patients with type 2 diabetes, coronary artery disease or both conditions compared with healthy controls [10]. Although both studies share the limitations that patients were older than controls and that comparisons between groups were also not adjusted for confounders, they are interesting because of the age of the study samples. Mean ages of the different case and control groups were between 42 and 66 years. Thus, the absence of the initially hypothesised inverse association between systemic IL-22 levels and type 2 diabetes in our study may not be attributable to the advanced age of the KORA F4 study population.

The aforementioned data were corroborated by mechanistic studies indicating that IL-22 induced insulin resistance in human hepatocytes and rat skeletal muscle cells. However, these effects were achieved using in vitro concentrations that were ≥1000-fold higher compared to serum levels [7], so that the physiological relevance of this observations remains unclear. In contrast, IL-22 was also found to protect human endothelial cells from glucose- and lysophosphatidylcholine-induced injury, thus supporting atheroprotective effects [10].

Importantly, the associations between IL-22 and cardiometabolic risk factors did not translate into an increased diabetes risk in the longitudinal part of our study. This result may appear unexpected given the studies in mice indicating diabetes-protective effects. Blocking IL-22 signaling resulted in unfavourable effects on body weight, glucose tolerance and insulin sensitivity [4], whereas treatment with recombinant IL-22 proteins or IL-22 overexpression counteracted weight increase and improved hyperglycaemia, insulin resistance and inflammation [4, 5, 22]. Incubation of murine and human islets with IL-22 (50 ng/ml) reduced oxidative and endoplasmatic reticulum stress [5]. However, deficiency of endogenous IL-22 did not lead to metabolic aberrations [4], and one study also failed to observe metabolic consequences of IL-22 overexpression [6].

The reason for the discrepancies may lie in the IL-22 concentrations that were reached in vivo or applied in vitro. IL-22 serum levels in mice were ≤20 pg/ml [6] and thus comparable to those in our study. Metabolic effects of IL-22 overexpression were observed in transgenic lines with circulating IL-22 levels of 4000–7000 pg/ml [22], but not in a line with serum IL-22 of 600 pg/ml [6], thus arguing for protective effects at extremely supraphysiological levels only [23]. In any case, our data consistently argue against the initially hypothesised *inverse* association between physiological serum levels of IL-22 and diabetes risk.

One possible interpretation of the divergent findings may be that circulating levels of IL-22 represent a biomarker for the systemic response against cardiometabolic risk factors. Although these factors need to be more precisely defined, major candidates include not only immunological stimuli, but also oxidative stress, endothelial dysfunction, dyslipidemia and further obesity-related metabolic disturbances that contribute to the close interrelationship between inflammation, type 2 diabetes and cardiovascular disease [10, 24–26]. However, such an upregulation does not appear to be sufficient to protect against the onset of cardiometabolic diseases, whereas higher, experimentally induced concentrations may be of therapeutic benefit. This explanation suggests similarities between IL-22 and IL-1RA, which also has diabetes- and atheroprotective effects, but higher circulating levels indicate a higher risk of type 2 diabetes and cardiovascular disease [25, 27, 28]. The fact that one of the aforementioned studies found elevated levels of IL-22 in groups with type 2 diabetes and coronary artery disease compared with healthy controls points into this direction [10]. Finally, it remains to be elucidated to what extent the interpretation of IL-22 levels as protective or pathological factor depends on the immunological context, i.e. on the presence of other cytokines and the overall immunological milieu.

### Strengths and limitations

The population-based design and the combination of cross-sectional and prospective analyses to identify associations of serum IL-22 with cardiometabolic risk factors and risk of type 2 diabetes are strengths of this study. Our results for incident type 2 diabetes were robust in several sensitivity analyses.

The main limitation is the sample size in the prospective analysis. However, we had a statistical power of 88.9 and 81.5% to detect an unadjusted OR per doubling of serum IL-22 of 0.8 and 1.2, respectively, at α = 0.05. Moreover, we studied older individuals of German descent, which limits the generalisability of our observations to younger populations and populations with non-European descent. Finally, we were not able to precisely quantify serum IL-22 levels in the whole study sample because almost one-third of serum samples yielding levels below the LOD of the assay selected for this study.

## Conclusion

In the population-based KORA F4 cohort, serum IL-22 was independently associated with male sex, current smoking, lower HDL cholesterol, lower eGFR and higher IL-1RA levels, which all represent risk factors of type 2 diabetes and/or diabetes-related complications. However, serum IL-22 was neither independently associated with glucose tolerance and diabetes status in a cross-sectional setting nor with risk of incident type 2 diabetes during a follow-up time of 7 years. Our data argue against the utility of IL-22 as specific biomarker for prevalent or incident type 2 diabetes in humans, but identify potential determinants of IL-22 levels which merits further research in the context of cardiovascular diseases.

## Additional file

**Additional file 1.** Additional tables and figure.
