# Supplementary material for: Serum levels of interleukin-22, cardiometabolic risk factors and incident type 2 diabetes: KORA F4/FF4 study
Source: Cardiovasc Diabetol. 2017 Jan 31;16:17. doi: 10.1186/s12933-017-0498-6 (PMC5282888; doi:10.1186/s12933-017-0498-6)
Supplement: Supplementary file 1 — Additional file 1. Additional tables and figure. [file 12933_2017_498_MOESM1_ESM.docx]

**Table S1. Drop-out analysis: baseline characteristics of the KORA F4 study participants stratified by participation in the follow-up KORA FF4 study**

| **Variable** | **Participation in FF4** | **No participation in FF4** | ***P*** |
| --- | --- | --- | --- |
| *n* | 641 | 466 |  |
| IL-22 (pg/ml) | 6.46 (1.95; 12.28) | 7.15 (1.95; 14.05) | 0.247 |
| Age (years) | 69.0 ± 4.9 | 72.0 ± 5.6 | <0.001 |
| Sex (% male) | 53.0 | 49.6 | 0.254 |
| BMI (kg/m²) | 28.4 ± 4.3 | 29.3 ± 4.7 | <0.001 |
| Fasting glucose (mmol/l) ^a^ | 5.47 ± 0.62 | 5.54 ± 0.83 | 0.157 |
| 2-h glucose (mmol/l) ^a^ | 6.97 ± 2.16 | 7.35 ± 2.48 | 0.013 |
| Fasting insulin (pmol/l) ^a^ | 28.8 (19.8; 48.0) | 32.4 (21.0; 56.3) | 0.326 |
| 2-h insulin (pmol/l) ^a^ | 323.7 (175.7; 520.1) | 331.8 (194.1; 548.4) | 0.066 |
| HOMA-IR | 1.16 (0.74; 1.99) | 1.24 (0.83; 2.49) | 0.261 |
| ISI (composite) (1/((mmol/l)x(pmol/l))) ^a^ | 17.5 (10.1; 29.2) | 16.0 (9.2; 25.6) | 0.282 |
| HbA1c (%) | 5.72 ± 0.58 | 5.87 ± 0.78 | <0.001 |
| HbA1c (mmol/mol) | 39 ± 6 | 41 ± 9 | <0.001 |
| Glucose tolerance status: NGT / IFG / IGT / IFG&IGT / ndT2D / T2D (%) | 42.4/20.4/9.1/10.1/6.1/11.9 | 35.0/16.5/10.9/12.5/7.1/18.3 | 0.008 |
| Hypertension (%) | 58.8 | 68.0 | 0.002 |
| Total cholesterol (mmol/l) ^b^ | 5.07 ± 0.81 | 5.17 ± 1.03 | 0.407 |
| LDL cholesterol (mmol/l) ^b^ | 2.99 ± 0.67 | 3.14 ± 0.85 | 0.101 |
| HDL cholesterol (mmol/l) ^b^ | 1.42 ± 0.34 | 1.37 ± 0.30 | 0.214 |
| Triglycerides (mmol/l) ^b^ | 1.33 (0.95; 1.83) | 1.38 (1.02; 2.03) | 0.774 |
| Use of lipid-lowering drugs (%) | 22.6 | 27.0 | 0.096 |
| eGFR (ml/min per 1.73m²) | 78.9 ± 13.8 | 73.1 ± 16.1 | <0.001 |
| eGFR <60 ml/min per 1.73m² (%) | 9.4 | 21.0 | <0.001 |
| Smoking (never/former/current) (%) | 51.5/41.5/7.0 | 50.0/41.6/8.4 | 0.663 |
| Physically active (%) | 56.0 | 41.6 | <0.001 |
| hs C-reactive protein (mg/l) | 1.34 (0.70; 2.72) | 1.96 (0.99; 4.02) | <0.001 |
| IL-6 (pg/ml) | 1.47 (1.03; 2.26) | 1.86 (1.31; 2.78) | 0.001 |
| IL-18 (pg/ml) | 320.0 (253.3; 414.0) | 316.5 (250.0; 422.3) | 0.697 |
| Tumour necrosis factor α (pg/ml) | 2.02 (1.46; 2.98) | 2.01 (1.47; 2.86) | 0.181 |
| IL-1 receptor antagonist (pg/ml) | 293.6 (226.3; 383.7) | 332.1 (257.9; 440.8) | <0.001 |
| Soluble intercellular adhesion molecule 1 (ng/ml) | 229.9 ± 52.5 | 248.1 ± 63.5 | <0.001 |
| Adiponectin (µg/ml) | 10.0 (6.4; 15.2) | 10.2 (6.8; 15.1) | 0.115 |

Data are given as mean ± SD, median and 25^th^; 75^th^ percentiles or percentages, unless indicated otherwise. The *P* values are unadjusted. eGFR, estimated glomerular filtration rate; hs, high-sensitivity; IFG, impaired fasting glucose; IGT, impaired glucose tolerance; ISI, insulin sensitivity index; ndT2D, newly diagnosed type 2 diabetes; NGT, normal glucose tolerance; T2D, known type 2 diabetes. ^a^ Individuals with known type 2 diabetes excluded (*n*=160). ^b^ Individuals using lipid-lowering drugs excluded (*n*=270).

**Table S2.** **Serum IL-22 levels and odds ratios (OR) with corresponding 95% CI for associations between serum of IL-22 (ln-transformed) and categories of glucose tolerance in the KORA F4 study (cross-sectional analysis)**

| **Category of glucose tolerance (*n*)** | **IL-22 (pg/ml)** | **OR (95% CI) Model 1** | **OR (95% CI) Model 2** | **OR (95% CI) Model 3** | **OR (95% CI) Model 4** |
| --- | --- | --- | --- | --- | --- |
| NGT (*n*=435) | 6.19 (1.95; 12.71) | 1 | 1 | 1 | 1 |
| IFG (*n*=208) | 7.41 (1.95; 13.28) | 1.04 (0.87; 1.24) | 1.07 (0.89; 1.28) | 1.02 (0.85; 1.23) | 1.00 (0.83; 1.21) |
| IGT (*n*=109) | 5.99 (1.95; 13.05) | 0.93 (0.74; 1.17) | 0.97 (0.77; 1.22) | 0.94 (0.75; 1.19) | 0.94 (0.74; 1.19) |
| IFG/IGT (*n*=123) | 6.74 (1.95; 13.98) | 0.97 (0.78; 1.21) | 1.02 (0.82; 1.27) | 0.96 (0.77; 1.21) | 0.95 (0.76; 1.21) |
| Newly diagnosed type 2 diabetes (*n*=72) | 7.27 (1.95; 11.57) | 0.99 (0.76; 1.30) | 1.03 (0.78; 1.35) | 0.96 (0.73; 1.27) | 0.91 (0.68; 1.23) |
| Known type 2 diabetes (*n*=160) | 8.77 (4.15; 14.81) | 1.13 (0.93; 1.37) | 1.13 (0.93; 1.38) | 1.02 (0.82; 1.26) | 0.94 (0.75; 1.18) |

NGT, normal glucose tolerance; IFG, impaired fasting glucose; IGT, impaired glucose tolerance. IL-22 serum levels are given as median (25^th^; 75^th^ percentiles) and were ln-transformed for the multinomial logistic regression analysis.

Model 1: adjusted for age and sex. Model 2: model 1 + smoking, alcohol consumption, physical activity. Model 3: model 2 + BMI. Model 4: model 3 + HDL cholesterol, LDL cholesterol, triglycerides, hypertension, prevalent myocardial infarction, eGFR.

**Table S3.** **Serum IL-22 levels and odds ratios (OR) with corresponding 95% CI for associations between serum of IL-22 (ln-transformed) and prediabetes and type 2 diabetes in the KORA F4 study (cross-sectional analysis)**

| **Diabetes status (*n*)** | **IL-22 (pg/ml)** | **OR (95% CI) Model 1** | **OR (95% CI) Model 2** | **OR (95% CI) Model 3** | **OR (95% CI) Model 4** |
| --- | --- | --- | --- | --- | --- |
| NGT (*n*=435) | 6.19 (1.95; 12.71) | 1 | 1 | 1 | 1 |
| Prediabetes (*n*=440) | 6.89 (1.95; 13.28) | 0.99 (0.86; 1.15) | 1.03 (0.89; 1.19) | 0.99 (0.85; 1.14) | 0.98 (0.84; 1.14) |
| Type 2 diabetes (*n*=232) | 7.85 (4.00; 14.20) | 1.08 (0.91; 1.29) | 1.10 (0.92; 1.31) | 1.00 (0.83; 1.21) | 0.93 (0.76; 1.15) |

NGT, normal glucose tolerance; prediabetes, all individuals with impaired fasting glucose and/or impaired glucose tolerance; type 2 diabetes, all individuals with newly diagnosed or known type 2 diabetes. IL-22 serum levels are given as median (25^th^; 75^th^ percentiles) and were ln-transformed for the multinomial logistic regression analysis.

Model 1: adjusted for age and sex. Model 2: model 1 + smoking, alcohol consumption, physical activity. Model 3: model 2 + BMI. Model 4: model 3 + HDL cholesterol, LDL cholesterol, triglycerides, hypertension, prevalent myocardial infarction, eGFR.

**Table S4.** **Baseline characteristics of the study population in KORA F4 stratified by incidence of type 2 diabetes (prospective analysis from KORA F4 to KORA FF4)**

| **Variable** | **No incident T2D** | **Incident T2D** | ***P*** |
| --- | --- | --- | --- |
| *n* | 428 | 76 |  |
| IL-22 (pg/ml) | 6.28 (1.95; 12.35) | 6.45 (1.95; 11.80) | 0.744 |
| Age (years) | 68.6 ± 4.9 | 68.8 ± 4.9 | 0.771 |
| Sex (% male) | 50.9 | 56.6 | 0.369 |
| BMI (kg/m²) | 27.4 ± 3.7 | 30.3 ± 4.7 | <0.001 |
| Fasting glucose (mmol/l) | 5.30 ± 0.47 | 6.00 ± 0.51 | <0.001 |
| 2-h glucose (mmol/l) | 6.35 ± 1.53 | 8.08 ± 1.67 | <0.001 |
| Fasting insulin (pmol/l) | 26.4 (18.6; 43.2) | 40.5 (25.65; 76.35) | 0.002 |
| 2-h insulin (pmol/l) | 276.9 (146.7; 423.9) | 488.7 (332.7; 772.1) | <0.001 |
| HOMA-IR | 1.03 (0.71; 1.68) | 1.74 (1.09; 3.56) | <0.001 |
| ISI (composite) (1/((mmol/l)x(pmol/l))) | 20.2 (12.5; 34.5) | 10.7 (6.0; 14.7) | <0.001 |
| HbA1c (%) | 5.51 ± 0.28 | 5.82 ± 0.32 | <0.001 |
| HbA1c (mmol/mol) | 37 ± 3 | 40 ± 4 | <0.001 |
| Glucose tolerance status: NGT / IFG / IGT / IFG&IGT (%) | 59.3/23.4/10.0/7.2 | 3.9/36.8/17.1/42.1 | <0.001 |
| Hypertension (%) | 50.5 | 71.1 | 0.001 |
| Total cholesterol (mmol/l) ^a^ | 6.02 ± 1.04 | 5.85 ± 0.82 | 0.281 |
| LDL cholesterol (mmol/l) ^a^ | 3.87 ± 0.95 | 3.75 ± 0.68 | 0.386 |
| HDL cholesterol (mmol/l) ^a^ | 1.51 ± 0.38 | 1.34 ± 0.32 | <0.001 |
| Triglycerides (mmol/l) ^a^ | 1.23 (0.89; 1.62) | 1.49 (1.18; 1.98) | 0.035 |
| Use of lipid-lowering drugs (%) | 20.6 | 17.1 | 0.457 |
| eGFR (ml/min per 1.73m²) | 79.6 ± 13.7 | 79.5 ± 12.5 | 0.937 |
| eGFR <60 ml/min per 1.73m² (%) | 7.7 | 7.9 | 0.984 |
| Smoking (never/former/current) (%) | 51.9/40.7/7.5 | 60.5/36.8/2.6 | 0.085 |
| Physically active (%) | 60.7 | 47.4 | 0.036 |
| hs C-reactive protein (mg/l) | 1.32 (0.68; 2.55) | 1.53 (0.78; 3.28) | 0.603 |
| IL-6 (pg/ml) | 1.38 (0.98; 2.06) | 1.51 (1.09; 2.28) | 0.786 |
| IL-18 (pg/ml) | 341.9 ± 163.6 | 348.7 ± 137.0 | 0.894 |
| Tumour necrosis factor α (pg/ml) | 2.01 (1.44; 2.9) | 2.18 (1.63; 3.05) | 0.465 |
| IL-1 receptor antagonist (pg/ml) | 302.2 ± 129.9 | 358.8 ± 152.7 | 0.001 |
| Soluble intercellular adhesion molecule 1 (ng/ml) | 227.0 ± 51.7 | 233.3 ± 53.2 | 0.329 |
| Adiponectin (µg/ml) | 10.6 (7.4; 16.0) | 7.4 (5.6; 12.4) | <0.001 |

Data are given as mean ± SD, median and 25^th^; 75^th^ percentiles or percentages, unless indicated otherwise. The *P* values are derived from logistic regression analysis (likelihood ratio tests comparing models with the respective variable, age and sex as independent variables to models with age and sex only). All analyses were adjusted for age and sex except associations with age (sex-adjusted only) or sex (age-adjusted only).

eGFR, estimated glomerular filtration rate; hs, high-sensitivity; IFG, impaired fasting glucose; IGT, impaired glucose tolerance; ISI, insulin sensitivity index; NGT, normal glucose tolerance; T2D, type 2 diabetes. ^a^ Individuals using lipid-lowering drugs excluded (*n*=101).

**Table S5.** **Associations between serum levels of IL-22 and incident type 2 diabetes (OR and 95% CI) in the KORA F4/FF4 study: main analysis and sensitivity analyses**

| **Analysis** | **Model 1** | **Model 2** | **Model 3** | **Model 4** |
| --- | --- | --- | --- | --- |
| Main analysis: results for a doubling in serum levels of IL-22 | 1.02 (0.84; 1.23) | 1.04 (0.86; 1.26) | 1.01 (0.83; 1.24) | 1.03 (0.83; 1.27) |
| Sensitivity analysis 1: analysis of the original dataset before replacement of outliers with IL-22 levels corresponding to the 99th percentile | 1.03 (0.85; 1.25) | 1.03 (0.85; 1.25) | 1.01 (0.82; 1.24) | 1.01 (0.81; 1.25) |
| Sensitivity analysis 2: analysis of the data treating IL-22 as dichotomous exposure (IL-22 levels above versus below the LOD) | 1.06 (0.61; 1.85) | 1.11 (0.63; 1.96) | 0.99 (0.55; 1.78) | 1.01 (0.55; 1.85) |
| Sensitivity analysis 3: main analysis restricted to current non-smokers. | 1.03 (0.86; 1.24) | 1.02 (0.84; 1.23) | 1.01 (0.82; 1.24) | 1.02 (0.82; 1.26) |

Model 1: adjusted for age and sex. Model 2: model 1 + smoking*, alcohol consumption, physical activity. Model 3: model 2 + BMI. Model 4: model 3 + HDL cholesterol, LDL cholesterol, triglycerides, hypertension, prevalent myocardial infarction, eGFR.

*In sensitivity analysis 3, adjustment only for former smoking.

**Figure S1. Distribution of serum levels of IL-22 (ln-transformed) in the KORA F4 cohort stratified by sex.**


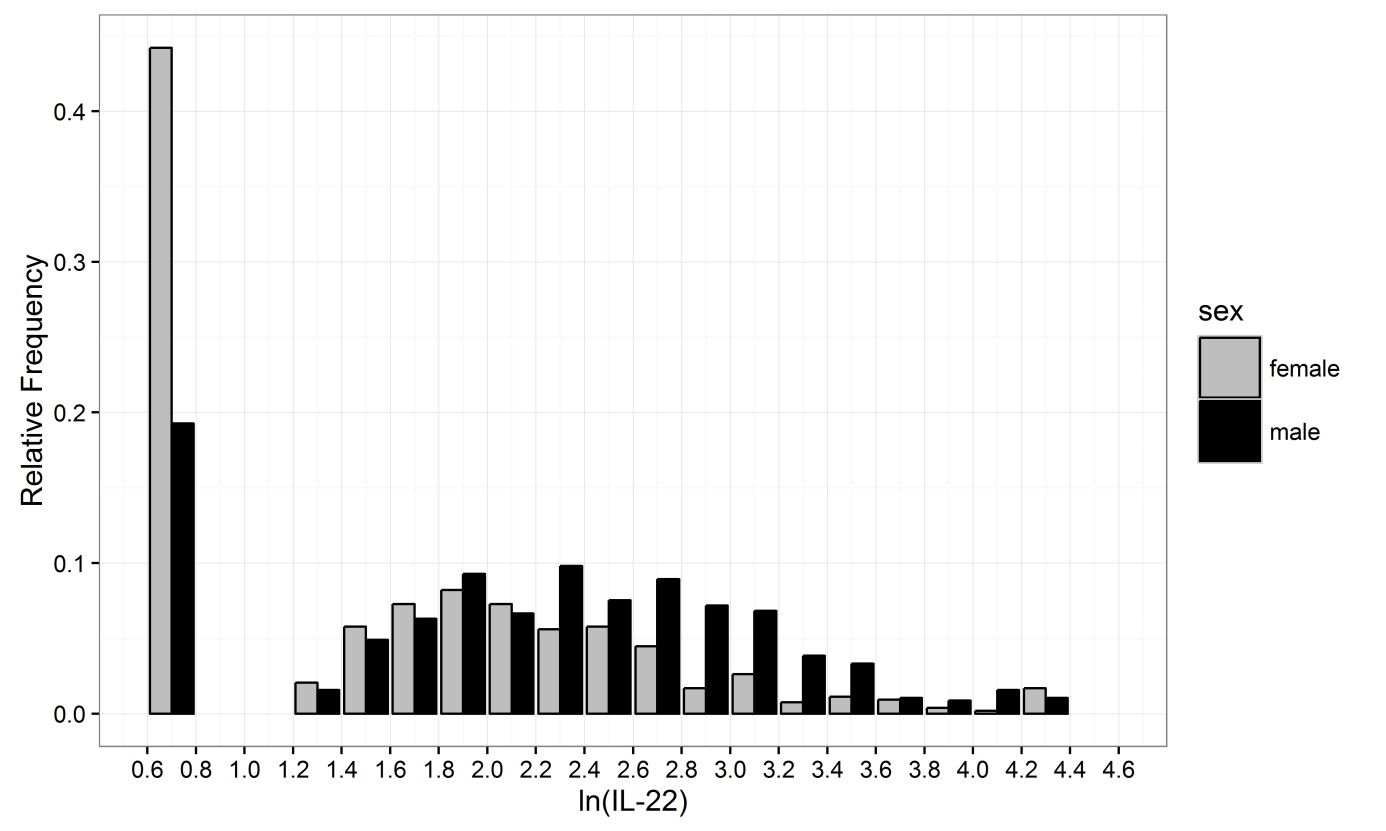


Values below the limit of detection (LOD, 3.9 pg/ml) were obtained for 19.3% of men and 44.2% of women. These serum samples were assigned a value of 1.95 pg/ml (=0.5*LOD). Values above the 99th percentile were assigned the IL-22 level of the 99th percentile (74.4 pg/ml).
